# Impact of Pregnancy and the Postpartum Period on Sudden Sensorineural Hearing Loss and Other Audiological Changes

**DOI:** 10.3390/jcm15166477

**Published:** 2026-08-21

**Authors:** Natalia Tomala, Paweł Fecica, Agata Ciećka, Julia Graca, Gabriela Dudek, Anna Tracz, Natalia Domaradzka, Daria Kluba, Karolina Dorobisz, Katarzyna Pazdro-Zastawny

**Affiliations:** 1 Students Scientific Club of Otolaryngology, Faculty of Medicine, Wroclaw Medical University, Ludwika Pasteura 1, 50-367 Wrocław, Poland; pawel.fecica@student.umw.edu.pl (P.F.); agata.ciecka@student.umw.edu.pl (A.C.); julia.graca@student.umw.edu.pl (J.G.); gabriela.dudek@student.umw.edu.pl (G.D.); anna.tracz@student.umw.edu.pl (A.T.); natalia.domaradzka@student.umw.edu.pl (N.D.); daria.kluba@student.umw.edu.pl (D.K.); 2 Department of Otolaryngology, Wroclaw Medical University, Borowska 213, 50-556 Wroclaw, Poland; karolina.dorobisz@umw.edu.pl (K.D.); katarzyna.pazdro-zastawny@umw.edu.pl (K.P.-Z.)

**Keywords:** sudden hearing loss, pregnancy, intratympanic steroid injections, vertigo, tinnitus

## Abstract

**Background/Objectives:** Pregnancy is associated with profound hormonal, hemodynamic, and metabolic changes that may affect the auditory and vestibular systems. However, evidence regarding pregnancy-related audiological disturbances remains limited due to the low incidence of these conditions and the scarcity of dedicated studies. The study aimed to identify the most commonly reported audiological changes occurring during pregnancy and the postpartum period with an emphasis on sudden sensorineural hearing loss (SSNHL), its risk factors, clinical presentation, and treatment methods. **Methods:** A literature search was conducted in PubMed, Embase, Web of Science, and Google Scholar from database inception to November 2025 using combinations of the keywords “pregnancy”, “hearing loss”, and “audiological changes”. The review is based on twenty-four original articles that met the preselected criteria. **Results:** Current evidence does not support pregnancy as an independent risk factor for SSNHL; the prevalence in obstetric patients is lower than in the control groups. Most cases occur during the third trimester or the postpartum period. Pre-pregnancy obesity and elevated blood pressure were identified as potential risk factors. Higher income levels and rural residency are associated with an increased incidence of SSNHL, whereas gestational diabetes, pre-eclampsia, and pregnancy-related weight gain showed no consistent association with SSNHL. Audiometric studies demonstrated mild, predominantly low-frequency, hearing threshold elevations that were generally transient and resolved after delivery. Tinnitus, nausea, aural fullness, dizziness, and vertigo were the most common ear-related complaints. Most of these disorders resolve spontaneously, but some studies suggest steroid treatment should be introduced. Intratympanic corticosteroid therapy was the most frequently investigated treatment for SSNHL and was associated with favorable hearing outcomes in most reported cases. Limited evidence suggests that Dextran 40 may improve hearing outcomes when used as an adjunctive therapy. **Conclusions**: Potentially transient cochleovestibular impairment during pregnancy and the postpartum period should not be underestimated and requires further evaluation. Pregnancy-related physiological adaptations may contribute to transient auditory and vestibular disturbances, particularly during late pregnancy and the postpartum period. Although current evidence does not indicate an increased risk of SSNHL during pregnancy, prompt recognition and management remain important to prevent long-term hearing impairment. Further high-quality studies are required to clarify the underlying mechanisms and establish evidence-based treatment recommendations for pregnant patients with SSNHL.

## 1. Introduction

Sudden sensorineural hearing loss (SSNHL) is clinically defined as a subjective unilateral decrease in hearing that occurs suddenly and occurrs within 72 h. The audiometric criteria define this disorder as a hearing loss of at least 30 decibels [dB] affecting a minimum of three contiguous frequencies. In the absence of previous audiometry measurements, the patient’s unaffected ear is utilized as the reference for comparison whenever possible [1]. Bilateral SSNHL represents a distinct pathophysiological process and should be managed as a medical emergency., as it may be an acute manifestation of the severe underlying disease [2,3]. Pure-tone audiometry is the first-line choice for SSNHL evaluation and recovery monitoring. Except that, auditory brainstem response testing (ABR), MRI or CT should be performed if a malignancy is suspected. Laboratory tests are indicated especially if the patient presents with risk factors for the underlying condition such as Lyme disease or syphilis. In cases of idiopathic etiology of SSNHL, lipid analysis and serum glucose should be included in the serologic testing, as their elevation in many affected patients was observed [4].

Although over the years numerous studies have investigated the etiology of SSNHL, its underlying pathophysiological mechanisms remain incompletely understood. Multiple factors were identified to increase the risk of developing SSNHL, including emotional stress, toxins, obesity, hormonal and electrolyte level changes, trauma, and cancer. Acoustic trauma is defined as noise-induced sensorineural hearing loss and can be divided into acute and chronic types. Acute trauma is characterized by hearing impairment resulting from short-term exposure to high-intensity noise (>130 dB). Chronic acoustic trauma, in turn, known as noise-induced hearing loss (NIHL), develops from long-term exposure to a noisy environment [5]. Diabetes, autoimmune, neurological, metabolic, and cardiovascular diseases also predispose to this hearing disorder [6,7,8]. The latest hypothesis assumes intracochlear inflammation of idiopathic origin [9]. The first line of treatment of patients with SSNHL is systemic and/or intratympanic steroids [10].

The incidence of SSNHL in the Western population oscillates around 5 to 27 per 100,000 inhabitants annually, with the peak observed in individuals in their 60s and slightly higher prevalence in females [11,12,13]. Pregnant women account for only 0.3–3% of all SSNHL cases, which defines it as a rare pathology [11].

Pregnancy is associated with a wide range of physiological, hormonal, and hemodynamic changes that affect multiple organ systems, including the auditory system [14]. The cochlea is very susceptible to microcirculation disorders due to its reliance on terminal arterial supply and higher metabolic demand. Reduced deformability of erythrocytes and their increased aggregation are observed, as well as elevated plasma viscosity and increased fibrinogen levels. Hormonal changes occurring during pregnancy, particularly increased estrogen and progesterone levels, along with weight gain, can lead to fluid accumulation in the intercellular space and the development of edema. These hormonal fluctuations affect enzymatic processes and neurotransmitter activity, which may cause disruptions in the homeostasis of labyrinthine fluids [15]. Research suggests that changes in electrolyte concentrations, particularly changes in serum sodium concentration, may be a risk factor for the development of SSNHL, tinnitus, and severe hearing loss [16]. As a result of these physiological adaptations, pregnant women may report various auditory and vestibular symptoms, such as tinnitus, aural fullness, a sensation of ear pressure, dizziness, and vertigo [2,17]. Hearing loss significantly increases the risk of tinnitus, which can lead to impaired speech understanding, reduced quality of life, and the development of psychiatric symptoms. Furthermore, tinnitus occurring together with hearing loss significantly increases the risk of depression [18].

Estrogen peaks can affect not only the cochlea where the estrogen receptors are located, but also auditory processing within the central nervous system [19].

This review aimed to systematically evaluate the available literature on audiological disorders occurring during pregnancy and the postpartum period, with particular emphasis on SSNHL. SSNHL is not the most commonly occurring audiological pathology in the investigated group; however, there exists an abundant literature on this topic, including treatment methods, which eventually encouraged us to describe it in detail.

## 2. Materials and Methods

### 2.1. Search Strategy

**Figure 1:**
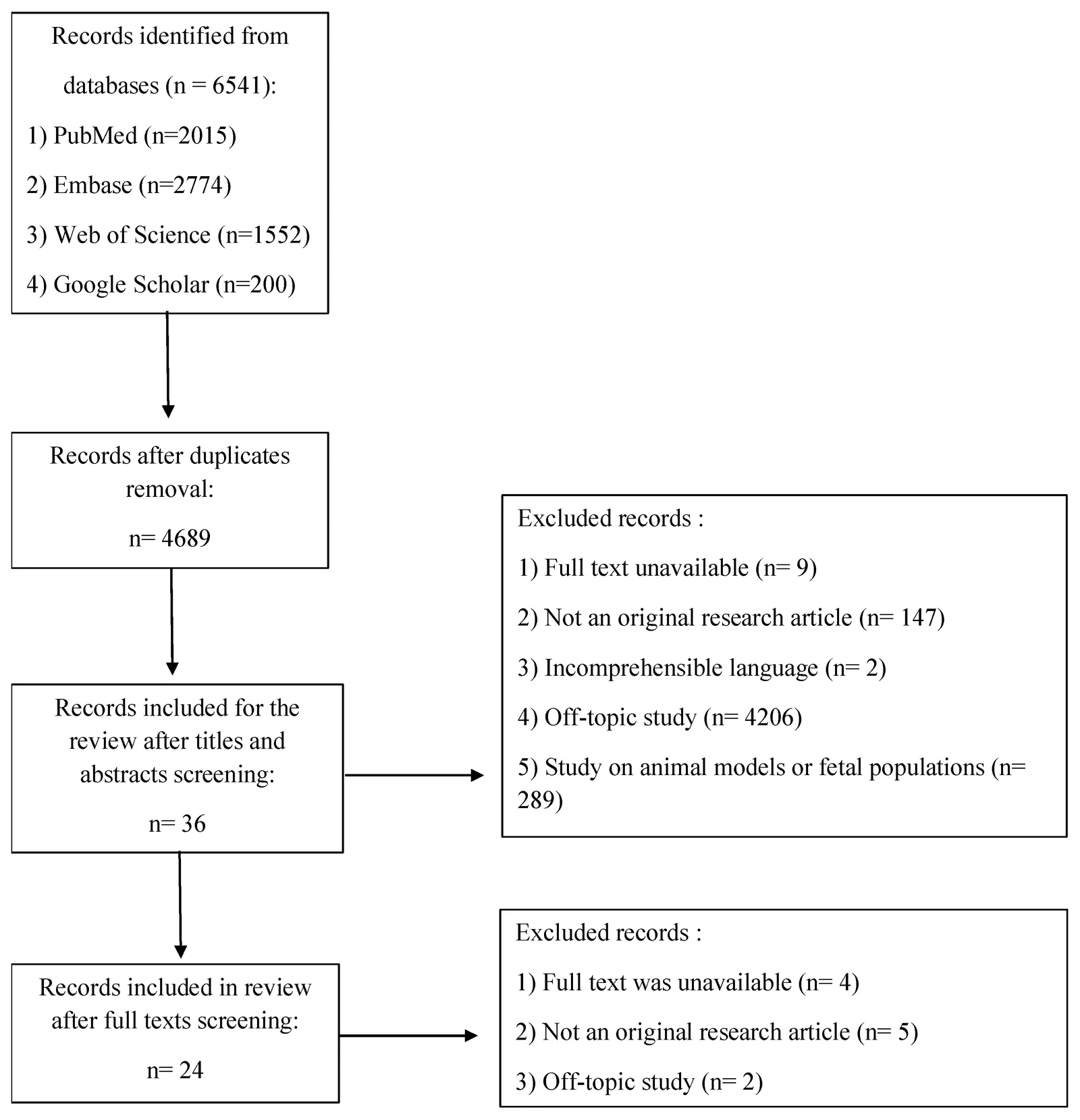
Study selection flowchart.

We searched the available medical literature for the audiological changes in pregnancy until November 2025. We searched PubMed, Embase, Web of Science and Google Scholar. All the results of these databases were reviewed without any publication date restrictions. We screened the first 200 results on Google Scholar, which is sufficient according to the literature. The search terms we used were “pregnancy” and “hearing loss” or “audiological changes”. We did not impose any language restrictions during the search. Six authors independently screened all the found articles using abstracts, titles, and keywords. The initial number of studies that resulted was 6541; then, 4689 remained after the deduplication process. The flowchart of study selection is presented in Figure 1.

### 2.2. Selection Criteria

Studies were included if they met all of the following criteria:

1. Original peer-reviewed research articles;
2. Studies reporting audiological changes associated with pregnancy or the postpartum period;
3. Studies clearly describing their objectives, methodology, and results;
4. Studies conducted in human populations.

Studies were excluded if:

1. The full text was unavailable;
2. The publication was not an original research article (e.g., reviews, editorials, conference abstracts, or case reports, series of case reports if applicable);
3. The study did not provide extractable data relevant to the review objectives;
4. The article was not peer-reviewed;
5. The study involved animal models or fetal populations.

Six independent reviewers screened the whole texts of previously selected studies in a way that each paper was evaluated by two different reviewers. They classified these studies as included or rejected for the review, relying on the selection criteria described above. If any discrepancies were encountered, a third reviewer was consulted to make the final decision. Eventually, 24 studies were selected and underwent data extraction.

### 2.3. Data Extraction

Double extraction was performed by six authors and then evaluated by another checking author. The primary data we collected were title, first author, publication year, and the type of study. More precise data we searched for included information on sample size, race, participants’ age range, gestational age, gravida status, symptoms, performed medical exams and frequencies used during the audiological tests, changes in audiological parameters, control group characteristics, follow-up period, applied treatment and its outcomes, cause or hypothesis for the audiological changes, final result or conclusion stemming from the study. The checking author resolved any discrepancies. One third of the included studies reported on SSNHL, seven on its treatment methods, four on pre-eclampsia, and five on other audiological complaints or audiological changes in general.

### 2.4. Outcome Analysis

Gathered data permitted a concise synthesis of the information on the audiological changes occurring in pregnancy and in the postpartum period. Many articles selected for the review used similar methodologies and focused on similar parameters in audiological tests, which allowed for a reliable comparison of their findings.

## 3. Results

### 3.1. Clinical Characteristics and Epidemiology of SSNHL During Pregnancy

#### 3.1.1. Epidemiology of SSNHL During Pregnancy

A recent nationwide South Korean study showed a yearly rise in SSNHL incidence in the control group without a history of delivery, whereas the delivery group maintained lower rates [11]. The delivery group comprised females aged 15 to 49 who gave birth between January 2013 and December 2019, categorized under ICD10: version 2019 codes O80 through O84. Most cases occurred during pregnancy (65.44–73.59%), with the remainder presenting in the postpartum period (26.41–34.56%); the risk was lowest in the first trimester, showing a significantly lower hazard ratio of 0.07 compared to controls, with only 24.2% of pregnancy-onset cases occurring here, and peaked in the third trimester, where the majority (60.6%) of cases presented [11,14,20,21]. Several studies agreed that pregnancy does not increase the risk of SSNHL, as pregnant women were significantly less likely to develop SSNHL than the non-pregnant control group [11,14,20].

#### 3.1.2. Risk Factors and Proposed Mechanisms

Pre-pregnancy metabolic factors such as smoking history, serum Hb, glucose, fasting glucose, cholesterol, HDL-cholesterol, TG, AST, and ALT were taken into consideration, in addition to physical examination including blood pressure and waist circumference measurements, as they can affect microcirculation. During gestation, elevated estradiol and progesterone levels increase thrombogenic risk by inducing a hypercoagulable state—marked by elevated fibrinogen, increased plasma viscosity, reduced erythrocyte deformability, and concurrent activation of blood coagulation and fibrinolysis. This state can cause a vascular insult to the cochlea via sluggish blood flow or microthrombosis, leading to SSNHL. Pre-pregnancy obesity (BMI ≥ 25 kg/m^2^) was associated with a higher risk of SSNHL in pregnancy. A statistically significant negative association between blood pressure and SSNHL was observed, with women experiencing SSNHL having lower mean systolic (109.0 ± 10.00 vs. 110.00 ± 10.84 mmHg) and diastolic blood pressure (68.40 ± 7.82 vs. 69.19 ± 8.12 mmHg). On the other hand, a potential association between hearing loss and hypotension, gestational diabetes, uncontrolled blood sugar, pre-eclampsia, and weight gain during pregnancy turned out to be statistically insignificant [21].

The incidence of SSNHL increases with age, with the highest rates reported in pregnant individuals aged 30–39 years. In individuals under 20 and over 40, hearing loss occurs very rarely, but it may be related to a small number of pregnancies at this age, resulting in insufficient data in research. Higher income levels and rural residency are associated with an increased risk of SSNHL. These demographics may be more susceptible to the condition due to heightened perceptions of work-related pressure, sedentary lifestyles, and a higher prevalence of alcohol consumption [11,20].

**Figure 2:**
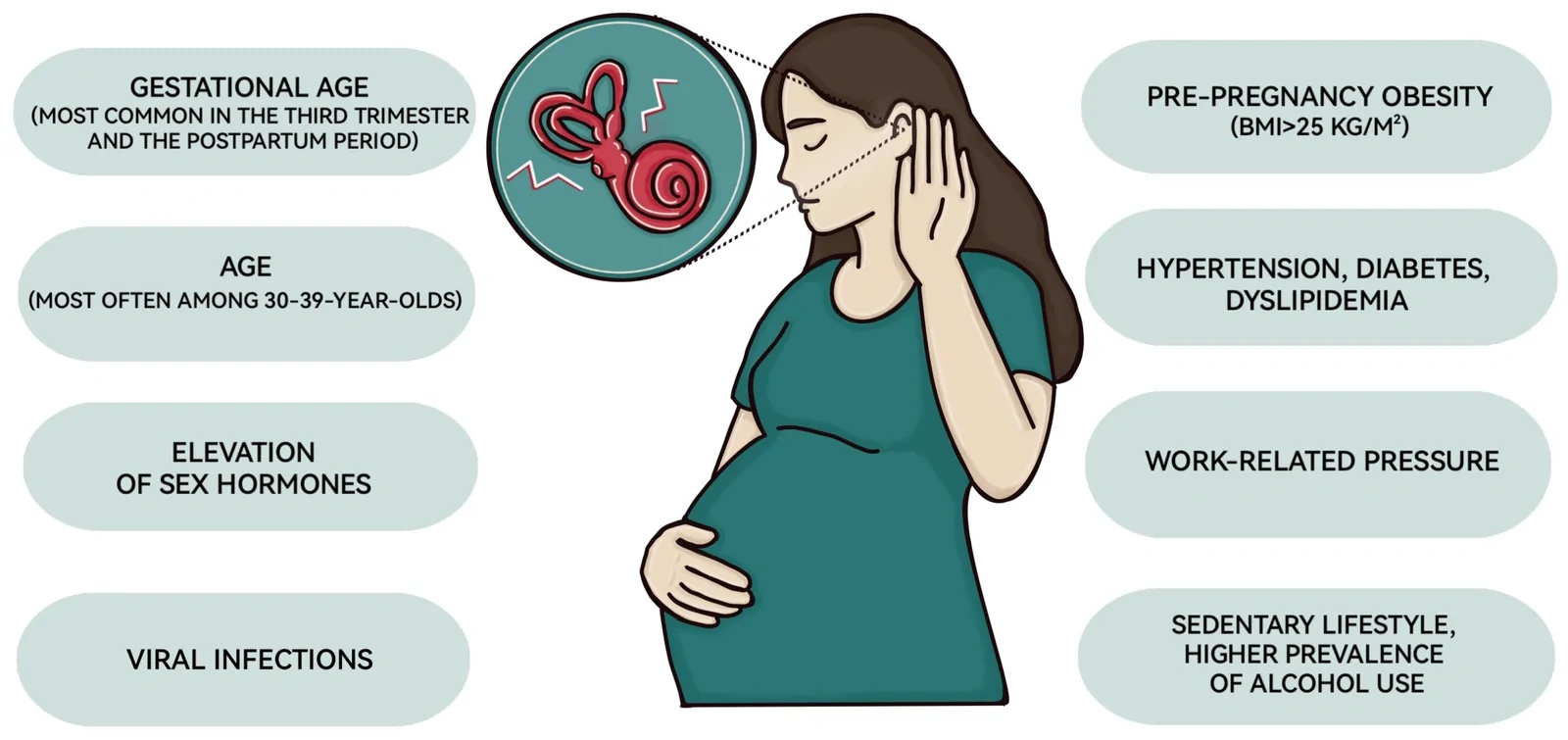
SSNHL risk factors during pregnancy. SSNHL—sudden sensorineural hearing loss; BMI—Body Mass Index.

**Figure 3:**
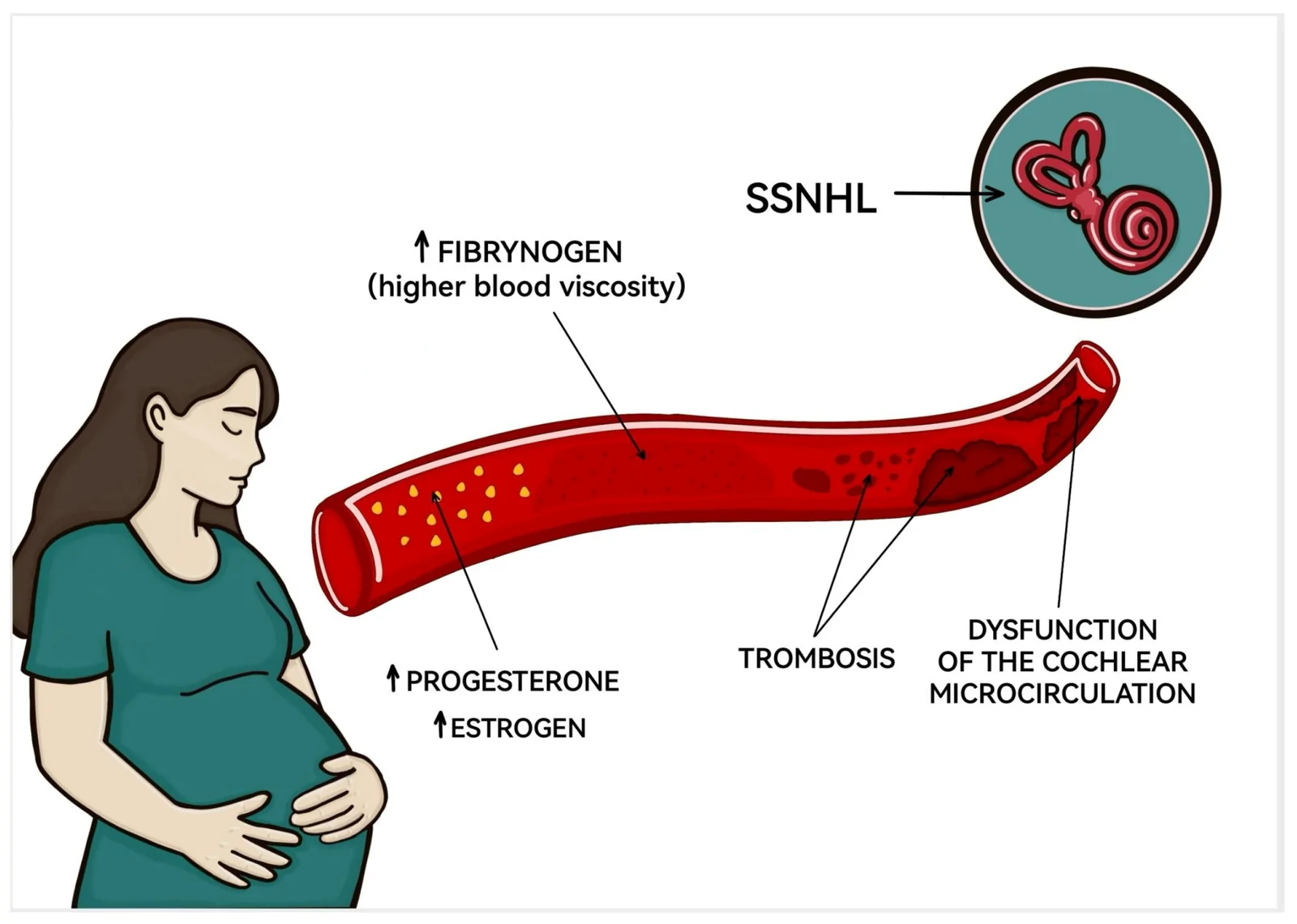
Microcirculation disorders in the cochlea due to hormone fluctuations during pregnancy. Uparrow indicates an increase in the concentration of a given substance in the blood.

Potential risk factors are presented in Figure 2 and the proposed mechanism for SSNHL development in Figure 3.

### 3.2. Treatment of SSNHL During Pregnancy

Most studies regarding the treatment of SSNHL focus on standard therapy—intratympanic steroids. The challenge is balancing efficacy and fetal safety. Therefore, treatments use the safest available drugs, following Food and Drug Administration (FDA) standards [22]. There are very few studies on SSNHL in pregnancy, so there is no single best way to treat it [8]. Treatment methods applied in the selected studies are gathered in Table 1.

#### 3.2.1. Corticosteroid Therapy

Currently, using corticosteroids is the main form of treatment. Steroids can be used alone or combined with other treatments. Studies varied in the route of administration, the choice of drug, the dose, and the duration of therapy.

- Steroid Mechanisms of Action in Sudden Sensorineural Hearing Loss

Systemic and intratympanic administration of corticosteroids constitute the main treatment approach used for SSNHL, despite not having clearly identified mechanisms of action [23]. The present literature reveals that steroids operate in a multipronged approach aimed at safeguarding cochlear functionality and minimizing damage caused by SSNHL. Regulating inner ear fluid dynamics with dexamethasone, one of the most used corticosteroids in intratympanic treatments, has been identified as playing an important role in sodium reabsorption and ion transport across inner ear tissues, which is crucial for fluid regulation. Modulation of vascular and microcirculation has been another theory regarding how corticosteroids affect the microvascular circulation of the cochlea in such a way that local ischemia is minimized, blood flow to the stria vascularis is increased, and protection from vascular injury occurs. When it comes to immunosuppression, by reducing inflammation and immunological processes that lead to tissue damage, including cellular edema, vascular changes, or autoimmune destruction of inner ear structures, steroids play a protective role [21].

- Intratympanic injections

Steroids were mostly given locally. The primary method was intratympanic administration. Since the steroid is injected directly into the middle ear, systemic absorption is minimal, reducing the risk of gestational diabetes or fetal growth issues. Dexamethasone is the most common drug used in this form [10,24,25].

There are minor differences among researchers regarding the dose and how often the drug is given. The results show a positive effect of the injections. However, the study samples were small, as sudden deafness is a rare condition.

In Yaoyao Fu’s study, 0.8 mL of dexamethasone [5 mg/mL] was given every other day for 7 days. Patients showed considerable hearing improvement, with a mean increase of 48.0 ± 7.33 dB [10].

Another study suggests four doses of dexamethasone [2.5 mg/0.5 mL] given every other day. After a week of therapy, the treatment group had a curative rate of 57.1%. They also showed significantly better hearing thresholds and more improvement than the control group. The injections caused no long-term side effects for the patients [24].

A difference in the postpartum treatment was observed. Patients were given a combination therapy of dexamethasone sodium phosphate injections [10 mg daily] along with Ginkgo biloba extract injection and mecobalamin injection. While this specific study did not explicitly define the injection route, other reviewed literature [8] indicates that Ginkgo biloba extract is typically administered intravenously in this clinical context. Additionally, during the postpartum period, lactation must be a significant consideration when selecting treatment options. All three patients showed better hearing levels across all frequencies that were tested. Moreover, the study established that despite 76.47% of patients presenting with profound or complete deafness, the pregnant group achieved superior hearing improvement and a significantly higher response rate (50%) compared to the non-pregnant group [26]. Researchers suggest that you can use methylprednisolone instead of dexamethasone. This drug can be used either as the main way of treatment or as a supplementary one [22,26].

- Postauricular injections

As a second, although much less common, option, some researchers suggest administering the drug via postauricular injection [8,26]. This method is mainly used if the patient refuses an intratympanic injection. The therapy involves a subperiosteal injection in the area of the upper half of the postauricular groove. The study from 2021 shows that a single 1 mL dose of betamethasone can also be administered in such a manner [8].

- Systemic steroid therapy

Systemic steroid therapy is another option mentioned in the literature, often as an alternative to local treatment [14]. In Yang et. al’s study, a systemic therapy with oral prednisone at a dose of 1 mg/kg/day was applied. Intratympanic injections proved to be effective as a salvage therapy if the systemic treatment does not work. Despite the initial discrepancies, there was no significant difference in effectiveness between pregnant and non-pregnant women with SSNHL. However, the degree of hearing improvement in pregnant women was worse than in the control group [22].

#### 3.2.2. Dextran Use

Another primary treatment for SSNHL patients involves intravenous dextran, often used together with steroids [25,27]. A study by Ming Xu describes a protocol where patients got intravenous infusions of 10% dextran-40 [500 mL] for 10 days. The group treated with additional intratympanic dexamethasone injections [0.4–0.6 mL of 5 mg/mL] turned out to present with remarkable effects. This group had better hearing thresholds (36.6 ± 19.4 vs. 47.2 ± 11.5 dB) and showed much more improvement (27.1 ± 16.4 vs. 15.7 ± 12.0 dB) compared to the group that only got dextran. However, there were no significant differences in other criteria. It suggests that we cannot undoubtedly assume that this combination is better than the standard treatment [25]. However, according to data in the antepartum pregnant group of patients, 88% of those treated with dextran in order to improve blood flow presented improvement, while only 12% of those who received no treatment recovered [27].

#### 3.2.3. Other Treatment Options

SSNHL is also treated with combination therapies using substances other than those mentioned above. One study suggests that glucose infusions can be combined with mecobalamin in treatment before delivery. In the control group of six patients, the response rate to the treatment was 33.33%. Adding intratympanic methylprednisolone led to much better clinical results. The response rate was 66.67%, with significant hearing recovery at frequencies 125, 250, 500, 4000, and 8000 Hz [26]. There is a lot of focus on the benefits of natural compounds, specifically therapies involving injections of G. biloba extract [8,26]. Additionally, one study suggests that using antioxidants, such as N-acetyl-L-cysteine (NAC), helps reduce damage to the ear structures. In one study, two postpartum patients taking oral NAC [600 mg twice daily for three months] improved their hearing from 110 dB to 52 dB and 28 dB [27]. However, there are currently no specific, large-scale studies investigating the effects of these natural compounds on auditory disorders specifically during pregnancy. Existing data comes only from very small groups where they were used as additional treatments, which remains a major limitation for drawing firm conclusions.

**Table 1:** Characteristics of studies describing hearing loss management in obstetric patients.

Study	Country	Study Design	Sample Size	Treatment Method	Drug [Dosage]	Therapy Duration	Treatment Results
Yaoyao Fu et al. (2018) [10]	China	Prospective study	6	ITS	Dexamethasone 0.8 mL [5 mg/mL]	On alternate days for 7 days	Improvement in hearing 48.0 ± 7.33 dB (measured by PTA at 500, 1000, 2000 and 4000 Hz)
Yan-Lu Lyu et al. (2020) [24]	China	Retrospective chart review	7	ITS	Dexamethasone [2.5 mg/0.5 mL]	4 doses on alternate days [total dose 10 mg]	55.2 ± 25.7 dB after one week of medical attendance; response rate 57.1%
Yi Qian et al. (2021) [8]	China	Retrospective chart review	15	ITS	Methylprednisolone 0.4 mL [40 mg/mL] and 0.1 mL of 2% lidocaine	5 times, once every 2 days	Response rate 53.3%; PTA improved from 103 ± 14 dB to 80 ± 29 dB
			3	PAS	Betamethasone 1 mL	Single subperiosteal injection	No detailed data available; 2 patients showed improvement, 1 showed no improvement (determined based on formal PTA assessments).
			2	OS + ITS	OS [methylprednisolone 40 mg]; ITS [methylprednisolone 0.4 mL [40 mg/mL] and 0.1 mL of 2% lidocaine	OS—3 days; ITS—standard protocol	No detailed data available; 1 patient showed improvement, 1 showed no improvement (determined based on formal PTA assessments).
			3	ITS + IvT	ITS [standard protocol]; Alprostadil [10 µg] Ginkgo leaf extract and dipyridamole [15 mg] and [Batroxobin 2/3 cases]	Alprostadil 10 µg once per day; Ginkgo leaf extract and dipyridamole: twice per day; Batroxobin: on alternate days until fibrinogen <0.5 g/L	No detailed data available; 2 patients showed improvement, 1 showed no improvement (determined based on formal PTA assessments).
Sen Yang et al. (2023) [22]	China	Prospective study	80	OS	Prednisone [1 mg/1 kg/1 day [max 50 mg]]	Full dose for 5 days	Combined data for all patients, n = 102: total effective rate of 28.4% (assessed by PTA)
			22	OS + ITS	OS [Prednisone, 1 mg/kg/day]; ITS 0.4–0.8 mL [Methylprednisolone 40 mg/mL or 30 mg/mL]	4 injections over a 2-week period	PTA of pre-treatment 84.45 ± 14.83 dB; PTA of 12 weeks after treatment 69.92 ± 17.91 dB;
Ming Xu et al. (2019) [25]	China	Retrospective observational study	16	IvT + ITS	10% Dextran-40 [500 mL a day] + Dexamethasone [0.4–0.6 mL of 5 mg/mL]	Dextran-40; 10 days; ITS—alternate days three times	Initial hearing threshold 64.5 ± 27.8 dB; Final hearing threshold 36.6 ± 19.4 dB;
			14	IvT	10% Dextran-40 [500 mL a day]	10 days	Initial hearing threshold 62.3 ± 22.6 dB; Final hearing threshold 47.2 ± 11.5 dB;
Bang-Yan Zhang et al. (2017) [27]	Taiwan	Retrospective cohort study	8	IvT	Dextran [1.0 L/day]	Total dosage of 3.5 L in 4 days	Mean hearing gain, 32 ± 16 dB; Response rate 88%
Xiao-Nan Wu et al. (2024) [26]	China	Retrospective study	6	IvT	Glucose [10%, 500 mL a day]+ Mecobalamin [0.5 mg a day]	7 days	IV—Response rate 33.33%; IV + PAS—Response rate 66.67% For all patients, n = 12: PTA of pre-treatment 102.81 ± 17.46 dB; PTA after treatment 88.02 ± 25.03 dB
			6	IvT + PAS	IV: Glucose [10%, 500 mL a day]+ Mecobalamin [0.5 mg a day]; ITS: Methylprednisolone [40 mg]	IV: 7 days ITS: on alternate days [3 times total]	

ITS—intratympanic steroid injection; OS—oral steroids; PAS—postauricular steroids; IV—intravenous; IvT—intravenous therapy; PTA—pure tone audiometry.

### 3.3. Pre-Eclampsia and Hearing Function

Pre-eclampsia is a systemic condition that affects multiple organs, such as kidneys, liver, central nervous system and heart. It is an effect of endothelial damage leading to proinflammatory cytokine release and coagulation cascade activation, reducing maternal systemic organ perfusion. It is a complication of 2–7% of pregnancies and is characterized by hypertension and proteinuria occurring after the 20th week of gestational age. Pre-eclampsia may cause increased cerebral blood flow, which contributes to vasogenic edema. Changes in cerebral blood flow provoke maldistribution and ischemia of the surrounding tissues, which may generate necrosis and hemorrhage. High pressure in the cochlear circulation might provoke hemorrhages in the inner ear as well as ionic changes in cell potentials of the hair cells, eventually causing progressive or sudden sensorineural hearing loss [28]. In pre-eclamptic patients, blood viscosity is increased, which contributes to decreased capillary blood flow and oxygen saturation becomes scarce. The symptoms that may appear are edema, visual or cerebral disturbances, headache, and epigastric pain [29,30,31]. Considering the pathogenesis of pre-eclampsia, a hypothesis that it can damage the inner ear epithelium was propounded [29]. Moreover, to investigate this theory, studies on pre-eclamptic patients were conducted.

Mohammed A. Gomaa et al. performed distortion-product otoacoustic emissions (DPOAEs). The background noise and the amplitude of response of the distortion product at 2 f1-f2 were retrieved at points corresponding to f2 frequencies of 553, 783, 1105, 1560, 2211, 3125, 4416, 6250, and 8837 Hz. This study showed significantly lower amplitudes at all tested f_2_ frequencies, except at 6250 Hz [31]. In another study, Transiently Evoked Otoacoustic Emission (TEOAE) was performed from 1500 to 4000 Hz band at 83 ± 3 dB intensity level. An amount of 5 of 37 patients in the study group revealed abnormalities that were consistent with hearing impairment, and the results were significantly different. All initial symptoms presented by patients fully recovered after 2 weeks [32].

Pure-tone audiometry provided evidence that the described condition can elevate hearing thresholds, especially at high frequencies [29,30].

Although the results turned out to be statistically significant, they were not clinically significant because all pure-tone audiometry thresholds were lower than 20 dB and patients did not present symptoms of hearing loss or deficit [29].

### 3.4. Other Audiological Changes During Pregnancy

Some pregnant women may experience mild and reversible changes in hearing sensitivity. Pure-tone audiometry data indicate that these changes are most evident in the lower-frequency range [33,34,35]. In third-trimester cohorts, air-conduction thresholds at 250 and 500 Hz were significantly higher in pregnant women than in non-pregnant controls [15]. Longitudinal data also show a gradual reduction in hearing acuity at 125–1000 Hz from the first to the third trimester, with stabilization in late pregnancy and return to baseline after delivery. Frequencies of 2000 Hz and above do not appear to change significantly, and no air-bone gap was observed [33,34]. Significant antepartum–postpartum differences in average air-conduction thresholds at speech frequencies have also been reported, with postpartum improvement in hearing sensitivity [33,34,35,36].

Subclinical cochlear changes may be present even when pure-tone thresholds remain within normal limits. Murthy and Krishna reported absent DPOAEs in a higher proportion of healthy pregnant women than non-pregnant controls despite normal pure-tone audiometry, suggesting possible outer hair cell dysfunction without overt threshold elevation [37].

Hyperemesis gravidarum presents with severe nausea, vomiting, and dehydration. Occurs only in 0.3–2% of pregnancies and is the most common reason for hospitalization in the first trimester [38]. As the vestibular system could be involved, the cochlear function was also checked for impairments. To examine the hearing in 29 affected patients, pure-tone audiometry testing at 250 and 500 Hz and 1, 2, 4, 8, 10, 12, 14, 16 kHz was performed. No differences were observed compared to healthy pregnant controls in tympanic membrane status and audiometric tests at any frequencies between [39]. Again, there were no differences observed between the group in tympanic membrane status on otoscopic examination and other otological evaluations including tympanometry.

The relationship between pregnancy and otosclerosis should be interpreted carefully. The available sources do not support presenting pregnancy as a proven etiological risk factor for otosclerosis. However, they do describe possible worsening of pre-existing otologic conditions during pregnancy, including otosclerosis, and note that women with otosclerosis may perceive hearing deterioration in the later months of pregnancy [35,40].

Among subjective otologic symptoms, tinnitus and vertigo appear to be the most frequently reported complaints during pregnancy. Schmidt et al. identified tinnitus as the leading auditory complaint in their cohort, followed by ear pressure/fullness and perceived hearing reduction. Questionnaire-based data discussed in the same article also indicate that symptoms such as ear fullness, tinnitus, and autophonia may occur during pregnancy and tend to subside after delivery [8,25,26,27,35,41].

The studies did not find evidence that the number of pregnancies affects the audiological changes during pregnancy, and they also did not observe any hearing deterioration at high frequencies [33,34].

Measures obtained in pregnant women have been reported as unremarkable, with peak compliance, peak pressure and gradient, ear canal volume, and acoustic reflexes all falling within normal limits and no difference in the condition of the tympanic membrane, and a separate cohort examined after 20 weeks same as showed in normal immittance and stapedial muscle reflexes in both pre-eclamptic women and controls [31]. However, multifrequency tympanometry across 250–2000 Hz differed: among 46 third-trimester women (92 ears) compared with 43 non-pregnant controls (86 ears), middle ear resonance frequency was significantly reduced and low-frequency hearing was poorer, findings the authors ascribed to edema of the middle ear mucosa arising from hormonal shifts and gestational weight gain [42]. Because peak middle ear pressure was reported in only one study, including patients at 9–11 weeks of pregnancy, no third-trimester pressure data are available against which the reported sensation of aural fullness might be assessed.The resonance frequency shift seen in late pregnancy was attributed to mucosal edema rather than to any measured change in pressure [39].

## 4. Discussion

Hormonal, metabolic, and anatomical transitions related to gestation may lead to the pathogenesis of many hearing disorders. Most studies associate the audiological impairments, which occur predominantly during the third trimester, with peak hormone levels affecting the sensorineural hearing system. Progesterone levels in the circulation reach levels 20 times higher, while serum estradiol levels are 30 to 40 times higher than in the normal menstrual cycle [43]. Although estrogen was considered to have a protective impact on hearing, it appears that high doses of estrogen or combined estrogen–progesterone therapy may trigger completely opposite responses from the auditory system. Such observations have already been made for hormone replacement therapy and oral contraception [44,45,46]. The aforementioned hormone fluctuations induce a hypercoagulable state that physiologically should reduce the risk of hemorrhage at delivery, but at the same time could compromise cochlear microcirculation. Moreover, cochlear circulation is easily compromised as it receives blood only through the labyrinthine artery and has poor collateral circulation. A recent study reports that SSNHL is frequently accompanied by vestibular dysfunction, which supports the concept of cochleovestibular vascular supply alterations [47].

What is more, comorbidities comprising hypertension, diabetes, abdominal obesity, and dyslipidemia were examined in terms of increasing susceptibility to SSNHL during pregnancy. The maternal metabolism shifts energy utilization from carbohydrate to fat, which is enabled by both increased insulin resistance and plasma concentrations of lipolytic hormones. That is why total serum cholesterol and triglyceride concentrations increase considerably during pregnancy, especially in the last months [39,48]. To our knowledge, studies investigating the metabolic aspect are scarce and include non-pregnant participants, so it was challenging to draw proper conclusions for this specific group of patients [6]. However, some researchers suggested that idiopathic SSNHL has a vascular etiology and therefore is strictly correlated with metabolic imbalances. Amongst the ischemic risk factors we can enumerate: cigarette smoking, hypertension, and hyperlipidemia [4,49]. Metabolic syndrome appears to be an independent risk factor for SSNHL and, simultaneously, a predictor of poor prognosis [50]. Elevated fasting glucose levels as well as glycosylated hemoglobin A1c have been shown to play a role in the atherogenic process and are thus a thrombogenic risk factor for the vulnerable inner ear circulation [51].

Estrogen plays a vital role in maintaining bone strength. It regulates bone metabolism by influencing bone structure and formation, and protects against resorption. Otosclerosis is a conductive hearing disorder affecting auditory ossicles that historically was mostly diagnosed in women [52]. Since its onset typically correlates with the reproductive period, it was speculated that estrogen signaling could be involved in stapedio-vestibular interface remodeling. Female hormones also stimulate pro-inflammatory reactions, potentially increasing pathologic bone turnover in the middle ear [53]. Hormonal fluctuations in late pregnancy and shortly after delivery make this period particularly prone to developing conductive hearing loss symptoms. We did not find evidence that pregnancy increases the risk for otosclerosis. However, we cannot exclude that hormonal changes are not associated with exacerbation periods, a phenomenon observed for other otological disorders [53,54,55]. Consequently, a greater number of pregnancies was hypothesized to negatively contribute to the level of severity and higher incidence of such disorders, yet from the reviewed papers we drew opposite conclusions. Quian’s study found that parenthood was associated with younger age at initial stapedectomy in patients with otosclerosis, but the effect was observed equally in both sexes [53]. In contrast, a British otosclerosis cohort supports the theory that women belong to a higher-risk group of developing clinical otosclerosis compared to men and that pregnancy may precipitate disease progression, at least in a proportion of women [56]. Interestingly, a 2022 study found that women showed better recovery of their middle ear function with better auditory thresholds and ABG after stapedectomy. The female hormones might improve chain flexibility by positively affecting the ligaments of the incudostapedial joint [57].

Symptoms were reported to resolve after delivery, when hormonal and fluid balance are returning to the pregestational state. However, some studies focus on the necessity of treating SSNHL to prevent permanent deafness. Whether sudden deafness should be treated remains controversial. Steroid therapy has been considered a gold standard, but in the literature we can find contradictory research findings. Several studies have demonstrated no significant benefit of steroid therapy compared with placebo [58,59]. Authors reported that the symptoms resolved after several months of follow-up [60]. The clinicians’ dilemma arises from the need to balance the potential risk of fetal exposure to harmful effects of administered medications against the necessity to preserve maternal hearing. Therefore, local treatment methods were preferred over systemic therapy, which was not the first-line management for non-pregnant patients. Our reviewed studies advised intratympanic steroids in the treatment of SSNHL along with adjuvant substances, as it also increased the probability of recovery. Response rates differed, but a 100% hearing improvement was never achieved even when additional substances such as dextran were applied. Dextran-40, a colloid plasma expander, is thought to reduce cochlear hypoxia by decreasing blood viscosity and improving microcirculation [44,61]. Nevertheless, volume expanders’ role has also been questioned with documented cases of dextran-40 causing pulmonary edema in patients with sudden deafness [62,63]. Treatment of other symptoms like tinnitus, nausea, aural fullness, pressure sensation, dizziness, or vertigo was not described as they are believed to be transient.

Unanimously, with other reviews, we did not find pregnancy to be a risk factor for SSNHL [64,65]. The incidence of SSNHL in the obstetric population is very low and does not appear to be higher than in the general population. The prevalence was observed to be either equal for both sexes or varied slightly depending on the study. A peak incidence occurs between ages 40 and 60, with most pregnancies occurring in 30-year-olds [27,66,67]. Its etiopathogenesis remains not fully understood; therefore, the majority of cases are considered idiopathic. Studies have identified viral infections, vascular disorders, and autoimmune mechanisms as potential causes [68]. If it comes to postpartum causes, apart from the hormonal interplay, other factors should be considered, such as viral infections facilitated by a weakened immune system in this period, superior semicircular canal dehiscence or stroke, potentially triggered by hypertension during pregnancy or increased intracranial pressure from excessive strain during labor [27].

Regarding the limitations of our review, only studies published in English were included due to the authors’ proficiency in foreign languages. Because of that, there is a chance some papers containing useful data were rejected during the screening stage because of this language criterion. We adopted a double-screening strategy to avoid omitting any eligible paper, but being performed by humans, the process carries a risk of error. A total of 4689 articles obtained from a search of four databases were deemed satisfactory; however, relevant studies may have been missed due to this database selection and excluding gray literature. Most of the included studies originated from East Asian countries, particularly China, South Korea, and Taiwan, which may limit the generalizability of the findings to other populations. Consequently, further research involving diverse ethnic and geographic populations is needed to provide a more comprehensive understanding of audiological changes during pregnancy and the postpartum period. An undeniable frequent bias of the selected studies was a small number of participants, which was due to the rarity of SSNHL. In addition, the studies were characterized by a certain heterogeneity and a lack of sufficient follow-up periods, which provides a suggestion for future research.

## 5. Conclusions

Complex changes in a woman’s body during pregnancy may affect the auditory system. Symptoms arise mostly in the third trimester and in the postpartum period and resolve spontaneously, sometimes without ever being noticed. Tinnitus and vertigo are the most common patients’ complaints. Mild decrease in hearing acuity at low frequencies has also been observed. There is no evidence that SSNHL occurs more frequently in pregnancy. Its treatment with steroids proved to be effective in the majority of cases. There is a need for more research to better understand the pathogenesis and to ascertain optimal guidelines for the management of this clinical condition.

## Data Availability

No new data were created or analyzed in this study. Data sharing is not applicable to this article.

## Abbreviations

The following abbreviations are used in this manuscript:

SSNHL	Sudden Sensorineural Hearing Loss
NAC	N-acetyl-L-cysteine
DPOAEs	Distortion-Product Otoacoustic Emissions
TEOAE	Transiently Evoked Otoacoustic Emission
ITS	Intratympanic Steroid Injection
OS	Oral Steroids
PAS	Postauricular Steroids
IvT	Intravenous Therapy
PTA	Pure Tone Audiometry
ABR	Auditory Brainstem Response testing
